# The use of augmented antibiotic-loaded cement spacer in periprosthetic joint infection patients with acetabular bone defect

**DOI:** 10.1186/s13018-020-01831-2

**Published:** 2020-10-01

**Authors:** Jun Fu, Yi Xiang, Ming Ni, Jiying Chen, Xiang Li, Baozhan Yu, Kan Liu, Yonggang Zhou, Libo Hao

**Affiliations:** 1 Department of Orthopaedics, Chinese People’s Liberation Army General Hospital (301 Hospital), Beijing, China; 2 Department of Orthopaedics, The 985 Hospital of PLA, Taiyuan, Shanxi China

**Keywords:** Augmented antibiotic-loaded cement spacer, Periprosthetic joint infection, Acetabular bone defect, Spacer complications

## Abstract

**Background:**

Spacer complications may affect final clinical outcome of the two-stage approach in periprosthetic joint infection (PJI) patients. This study aimed to investigate clinical outcomes and complications of augmented antibiotic-loaded cement spacer in PJI patients with acetabular bone defect.

**Methods:**

Data on PJI patients with acetabular bone defect receiving two-stage revision from January 2009 to December 2016, in our hospital were retrospectively reviewed. Screw-cement-shell was used to improve the stability of the hip with acetabular wall defect. Handmade acetabular spacer could prevent femoral spacer entering into pelvis in patients with acetabular internal wall defect. A total of 26 patients (11 males and 15 females) were included in the current study. Their mean age was 46.7 ± 15.4 years old. Clinical outcomes and complications were measured.

**Results:**

Twenty-one of total 26 hips (21/26, 80.8%) showed positive cultures and 15/26 (57.7%) samples were cultured with staphylococcus. Of enrolled patients, 5/26 (19.2%) developed mixed infection. There was one patient (3.8%) with spacer dislocation and two (7.7%) with spacer fracture. One patient developed acute PJI 5 years after the second-stage revision, so overall success rate among these patients was 96.2%. Differences in Paprosky classifications before the first and second stage did not reach significant level (*p* > 0.05). Hip Harris score was raised from 40.9 ± 14.0 to 81.2 ± 11.2 (*p* < 0.05).

**Conclusions:**

Augmented antibiotic-loaded cement spacer could achieve satisfactory clinical outcomes in PJI patients with acetabular bone defect. It provided joint mobility, increased additional joint stability, and decreased iatrogenic bone defect caused by acetabular wear.

## Introduction

Periprosthetic joint infection (PJI) remains one of the most severe and devastating complications in total hip arthroplasty (THA). Reportedly, the incidence rate of PJI among patients undergoing primary THA and revised THA was from 0.3 to 2.9% and from 0.95 to 22%, respectively [1–5]. There are several treatment methods for PJI, including prosthesis retention through debridement, one- or two-stage revision, resection arthroplasty, and arthrodesis. Despite resurgent interest in one-stage revision [6], two-stage revision is presently considered as the “gold standard” for PJI treatment, reaching a rate of infection clearance ranging from 87 to 93% [7–10].

After the removal of acetabular and femoral prosthetic components and thorough extensive debridement performed in the first stage revision, surgeon would be in a dilemma, adopting resection arthroplasty, or, much better, implanting antibiotic-loaded spacer whether static [11] or articulating [12]. Articulating spacers have the advantages of maintaining joint mobility, reducing scar formation, preventing soft tissue contracture, and enabling the patients to bear weights after surgery. However, it also has some potential disadvantages, like spacer dislocation, spacer fracture, peri-spacer fracture, and acetabular wear, especially for patients with acetabular bone defect, with reported complication rates between 13.2 and 58.8% [13, 14]. Spacer complications may cause prolonged hospitalization duration and sciatic nerve palsy, restrict the patients’ activities during the interim period before the second-stage revision, and have important effects on the final clinical outcome of the two-stage revision surgery.

Accordingly, we hypothesized that the application of augmented antibiotic-loaded cement spacer to temporarily repair acetabular bone defect could reduce spacer-related complications, and thus applied it in clinical practice. This current study aimed to report early clinical outcomes of this technique and to assess the consequent occurrence of complications and related risk factors.

## Materials and methods

After receiving approval from our institutional review board of our hospital (ChiCTR-INR-17013267), we retrospectively reviewed clinical records of PJI patients who underwent two-stage revision hip arthroplasty from January 2009 to December 2016. Inclusion criteria were (1) a definite diagnosis of periprosthetic hip infection according to the criteria proposed by MSIS in 2011 [15] diagnostic criteria were coincident for all hips; (2) undergoing two-stage revision; and (3) acetabular bone defect in the first-stage revision was treated with augmented antibiotic-loaded cement spacer. With any one of the following conditions, the patients would be excluded from the current study: (1) having severe complications, such as other bone diseases, inflammatory or immune diseases, systemic diseases, cardiovascular or cerebrovascular diseases, and tumors; (2) incomplete data sets; (3) a follow-up duration less than 12 months; (4) younger than 18 years; and (5) failing to receive two-stage revision.

Demographic characteristics of the patients were recorded, including age, sex, body mass index (BMI), interval duration before PJI, and diagnoses at the index operation. Microbiological data from pre- and intraoperative cultures were obtained. Radiological data were collected to evaluate the grade of acetabular bone defect according to Paprosky classification [16] and general mechanical complications (dislocation, fracture, etc.). All patients signed written informed consent to participate in the study and to give the permission to publish their clinical data and images.

Primary outcomes were clinical ones. Secondary outcomes contained spacer complication rates and successful rate of eradicating infection. A successful treatment outcome was defined as follows: (1) without any pathogenic microorganism on any culture specimen obtained from acetabular or femoral soft tissue and bone during the second-stage surgery; (2) no need for re-revision surgery caused by a recurrent or persistent infection of the involved hip during postoperative follow-up.

### Surgical technique

The patients adopted lateral position and posterolateral surgical approach was selected. Antibiotic administration was started only after at least 5-6 biopsy specimens (3 specimens at the acetabular bottom and femoral canal, respectively) for culture, and then the frozen section had been obtained. Major surgical procedures included extensive elimination of scars and sinus tract, radical debridement of all infected soft and bone tissues, and complete removal of both acetabular and femoral prosthetic implants. Our antibiotic-loaded cement protocol was to confect a total of 6 g of antibiotics per 40 g of cement (PALACOS® R + G, Zimmer Inc.). In a general way, sensitive antibiotics were determined via preoperative culture and antibiotic sensitivity test on infected joint fluid, when 4 g vancomycin and 2 g meropenem per 40 g of cement were routinely used. Typically, a total of 2-3 bags (80-120 g) of cement were used for every case.

### Screw-cement-shell

**Fig. 1:**
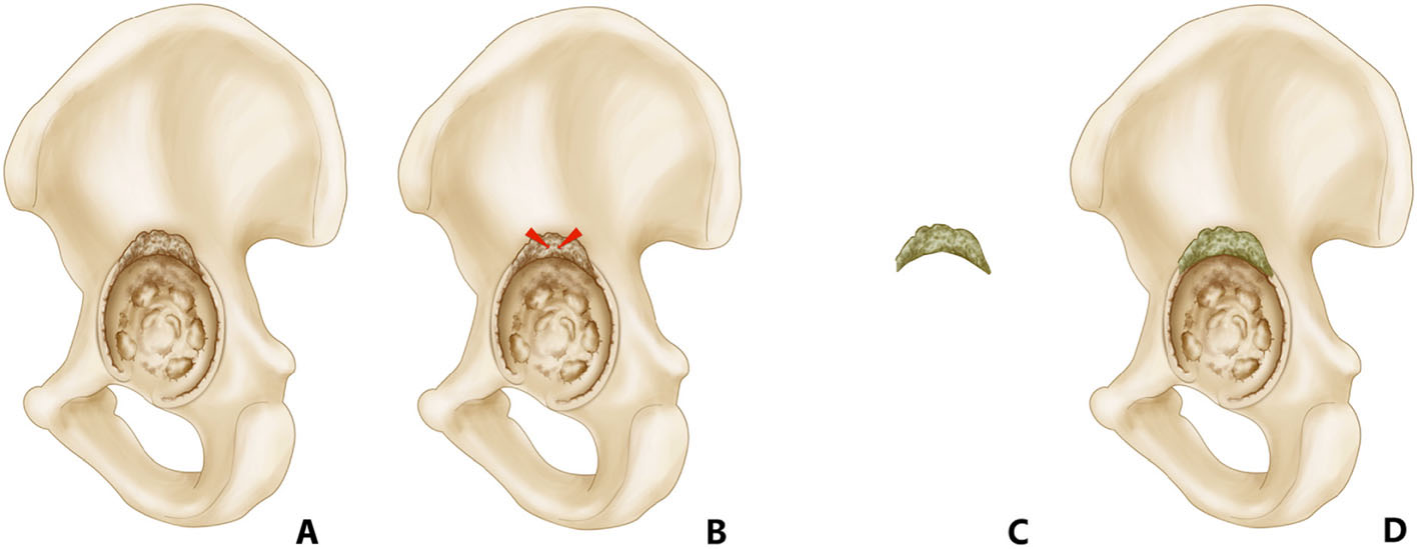
Schematic diagram of screw-cement-shell. **a** Superior wall of acetabular bone defect. **b** Two unicortical cancellous screws were inserted perpendicular to the surface of ilium, with approximately 20-25 mm of screw shaft remaining prominent. **c**, **d** Antibiotic-infused cement was placed around exposed part of the screws

Superior, inferior, anterior, and posterior walls of acetabular bone defects were temporarily repaired by screw-cement-shell to increase their stability. Firstly, the whole acetabular defect wall and a small supra-area were subperiosteally exposed to prevent surrounding vessels and nerves from damage. Furthermore, bony surface should be carefully visualized before implanting screw-cement-shell so as to prevent residual soft tissue from locating under screw-cement-shell, which might reduce mechanical performance of the screw-cement-shell. After bone surface was ready, two to three 3.5-mm unicortical drill holes were perforated with approximately 1 cm distance from the acetabulum, and then two to three 6.5-mm unicortical cancellous screws were implanted perpendicular to defect bone surface, with over approximatively 20-25 mm of screw tail being exposed. Antibiotic-loaded cement was placed around the exposed part of the screw nail and then the femoral spacer was inserted and stabilized within the femoral canal (Fig. 1).

### Handmade acetabular spacer

**Fig. 2:**
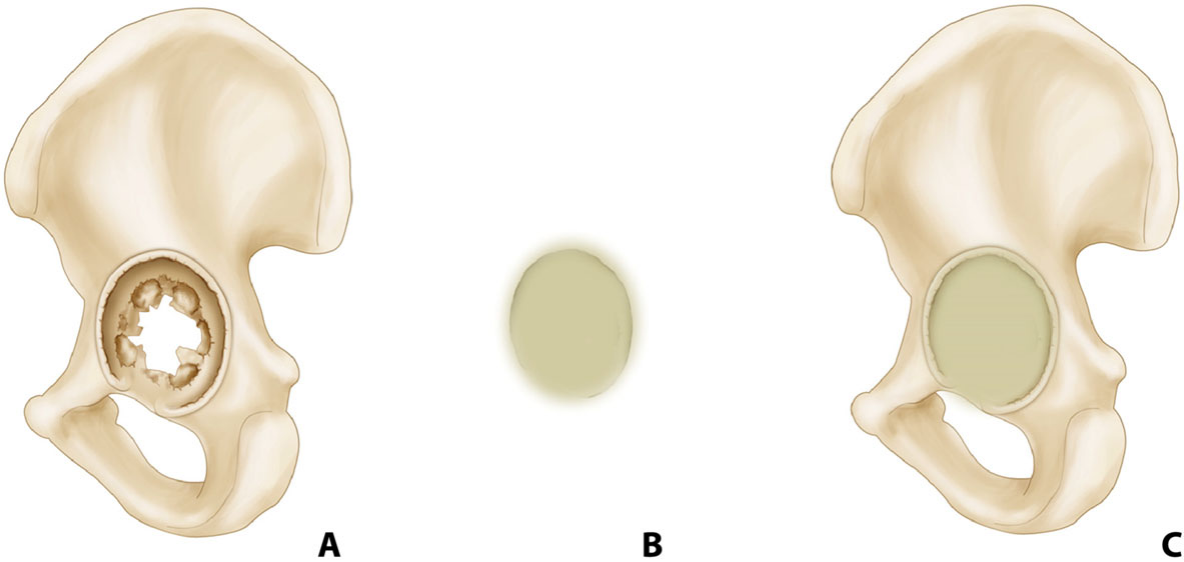
Schematic diagram of handmade acetabular spacer. **a** Medial wall of acetabular bone defect. **b** Handmade acetabular spacer was made during operation. **c** Acetabular spacer was placed to prevent femoral spacer into acetabulum

Medial wall of acetabular bone defect or severe acetabular protrusion was temporarily reinforced by handmade acetabular spacer. Handmade acetabular spacer was molded according to acetabular size, medial wall defect area, and the size of the femoral spacer head (Fig. 2). Acetabular spacer was then positioned and minimal pressure was applied, avoiding excessive infiltration of cement into the cancellous acetabular bone. This operation ensured easy removal of the acetabular spacer during the second-stage revision. Acetabular bone defects in major walls were fixed with cub-cage and antibiotic-loaded cement.

### Articulating femoral spacer

**Fig. 3:**
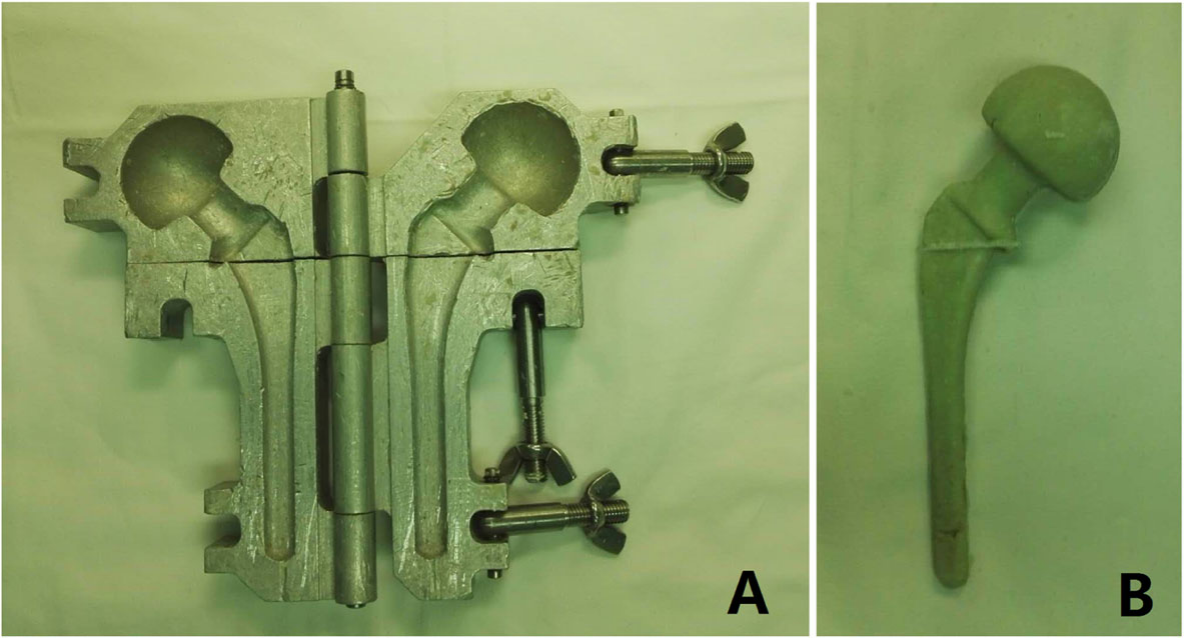
**a** Self-made spacer mold used in our study. **b** The spacer was made by self-made spacer mold

Articulating a femoral spacer was made with self-made spacer mold (Fig. 3), which has obtained an invention patent (CN201005806). The spacer mold has three different sizes. The size of the intraoperative mold was determined according to the femoral canal size measured by preoperative template. In general, one or two Steinmann pins were chosen as internal support of spacer and 60 g cement mixed with 9 g antibiotics was adopted to form femoral spacer using this mold.

Postoperatively, patients were encouraged to experience early rehabilitation through bearing partial weight from the first day after surgery. The patients were prescribed with intravenous antibiotic treatment for 4-6 weeks followed by oral antibiotics for another 6-8 weeks, based on cultured antibiotic sensitivity results obtained during the first-stage revision. In follow-up, patients’ clinical symptoms, laboratory examination (ESR and CRP), and radiological data were recorded; once wound healing and inflammatory mediators’ levels are satisfactory (CRP < 10 mg/L and ESR < 30 mm/h) [17], antibiotics would be discontinued. Reimplantation would be performed only when infection was ruled out through our evaluation. Our judgment standard for the second-stage revision included the disappearance of clinically infectious symptoms, normal inflammatory mediators, and an aspiration before surgery to rule out the recurrence of infection.

### Follow-up

Hip joint function of affected sites was estimated via Harris scoring system which was performed 8 weeks after the first and second revisions, respectively. Laboratory examination (ESR and CRP) and radiological detection were performed for postoperative infections and complications.

### Statistical analysis

Statistical analysis was conducted with SPSS 18.0 (SPSS Inc., Chicago, IL, USA) for windows. Continuous variables were recorded as mean and standard deviations when categorical variables were expressed as the number of cases and proportions. Comparisons between pre- and postoperative clinical parameters were completed adopting unpaired Student *t* tests or Wilcoxon rank-sum test. Categorical data were compared using chi-squared or Fisher’s exact tests. *P* value of < 0.05 was considered statistically significant.

## Results

A total of 26 eligible patients/hips (11 males and 15 females) who experienced two-stage revision surgery were included in the study, according to inclusion and exclusion criteria. Mean age was 46.7 ± 15.4 (ranging 18-75 years). The average BMI was 25.1 ± 4.3 (ranging 15.4-36.4 kg/m^2^). Interval duration between primary arthroplasty and confirmed PJI was 52.3 ± 54.6 (ranging 3-240 months). Diagnoses at the index operation with primary arthroplasty are shown in Table 1.

**Table 1:** Demographic and clinic features of the eligible patients

Features	Number of patients (%)
Age (years)	46.7 ± 15.4
Gender	
Male	11 (42.31)
Female	15 (57.69)
BMI (kg/m^2^)	25.1 ± 4.3
Acetabular bone defect type	
Avascular necrosis of femoral head	10 (38.46)
Femoral neck fracture	8 (30.77)
Hip osteoarthritis	4 (15.38)
Ankylosing spondylitis	3 (11.54)
Developmental dysplasia of hip	1 (3.85)
Microorganism	
Staphylococcus	15 (57.69)
Multiple organisms	5 (19.23)
Negative	5 (19.23)
Type of prosthesis fixation	
Screw-cement-shell	15 (57.69)
Handmade acetabular spacer	11 (42.31)
Follow-up time (years)	4.1 ± 2.2
First-stage (Paprosky)	
I	7 (26.92)
II	9 (34.62)
III	8 (30.77)
Second-stage (Paprosky)	
I	4 (15.38)
II	11 (42.31)
III	9 (34.62)
Hip Harris score (first stage)	40.9 ± 14.0
Hip Harris score (second stage)	81.2 ± 11.2
Interval duration between primary arthroplasty and confirmed PJI (months)	52.3 ± 54.6
Time between stages (months)	5.3 ± 3.7

*BMI* body mass index

Twenty-one of those 26 hips (21/26, 80.8%) showed positive cultures for pre- or intraoperative specimens taken during the first-stage. Staphylococcus appeared in 15 hips (15/26, 57.7%), and five hips (5/26, 19.2%) involved multiple organisms.

**Fig. 4:**
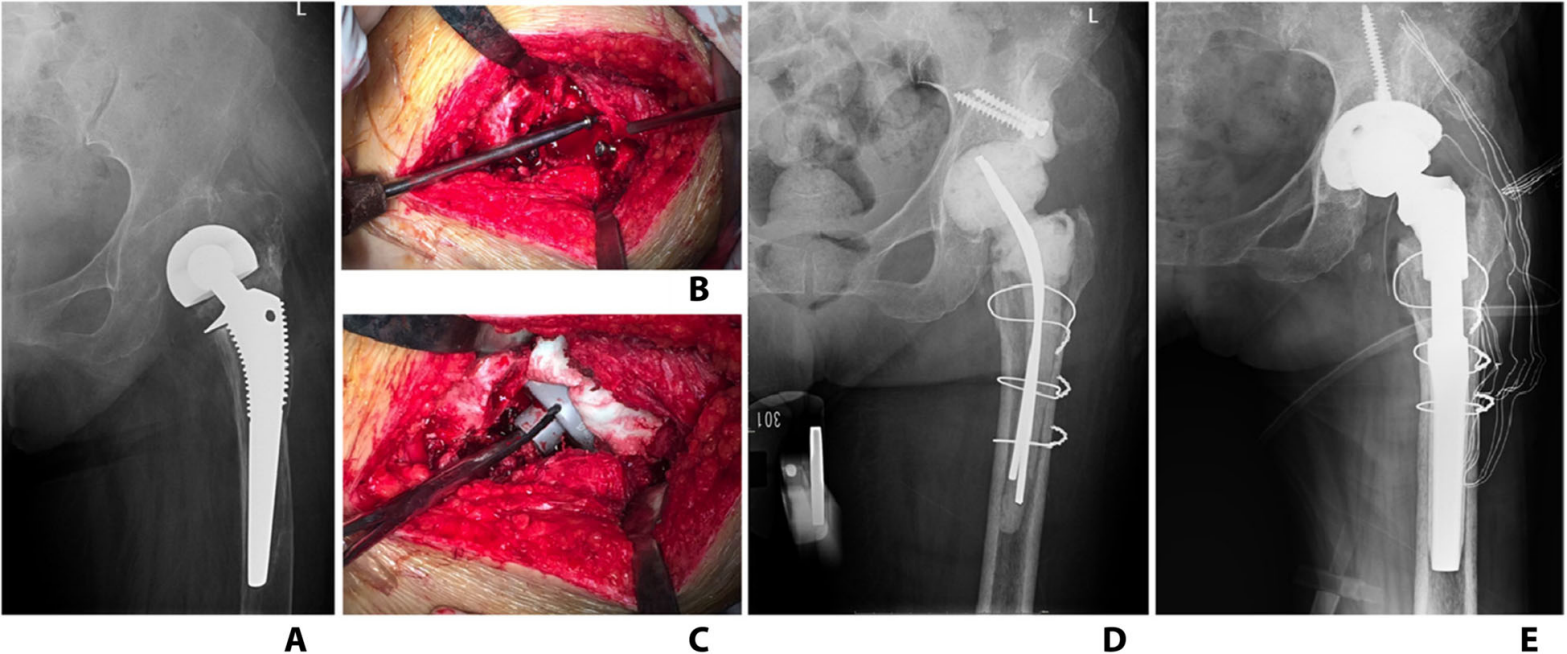
**a** One case with Paprosky IIB acetabular bone defect. **b**, **c** Screw-cement-shell was applied to reconstruct superior wall. **d** X-ray after spacer implantation. **f** X-ray after prosthesis implantation

**Fig. 5:**
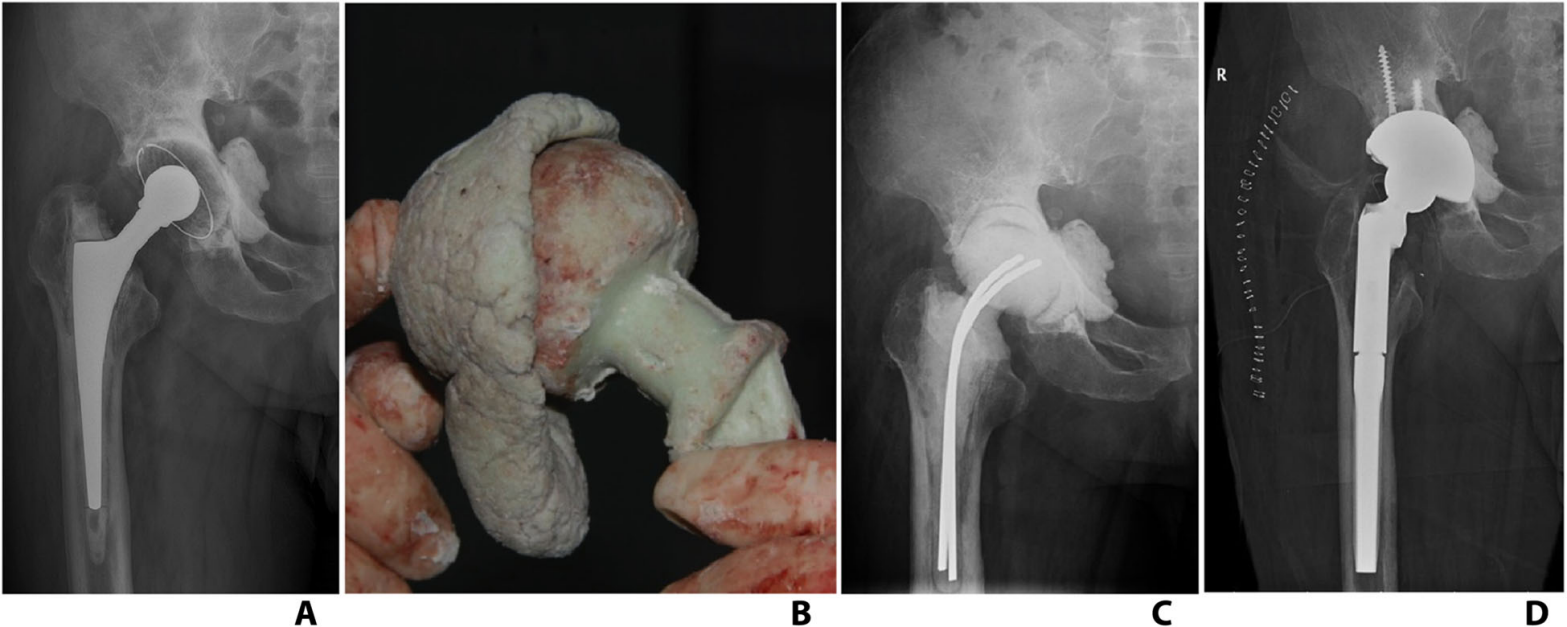
**a** One case with Paprosky IIC acetabular bone defect. **b** Handmade acetabular spacer was applied to reconstruct medial wall. **d** X-ray after spacer implantation. **f** X-ray after prosthesis implantation

Fifteen cases received reconstruction with screw-cement-shell (Fig. 4) and 11 cases with handmade acetabular spacer (Fig. 5).

Table 2 showed differences in Paprosky classifications (I, II, and III) before the first stage and second stage, without statistical significance (*p* > 0.05).

**Table 2:** Acetabular bone defects distribution according to Paprosky classification before the first stage and second stage

Paprosky stage	First stage	Second stage	*p* value
I	7	4	0.3029
II	9	11	0.5582
III	8	9	0.7628

Different local complications were checked during different periods. In the resection arthroplasty period, 4 patients (19.24%) presented local complications. A total of 34.36% of patients (9) occurred local complications in the reimplantation period and after reimplantation, a total of 10 cases (38.47%) presented different local complications. The detailed data was displayed in Table 3. No residual infection after the second surgery was found during the follow-up time.

**Table 3:** Local complications during different periods

Complications	Number of patients (%)
Resection arthroplasty period	
Persistent infection	3 (11.54)
Wound healing disorder	1 (3.85)
Bone fracture	1 (3.85)
Reimplantation period	
Blood loss	6 (23.08)
Bone fracture	1 (3.85)
New infection	1 (3.85)
Nerve palsy	1 (3.85)
After reimplantation	
Leg length discrepancy	3 (11.54)
Reinfection	2 (7.69)
Dislocation	1 (3.85)
Wound healing disturbance	2 (7.69)
Bone fracture	1 (3.85)
Aseptic loosening	1 (3.85)

During the follow-up, there was 1 case (3.8%) of femoral spacer dislocation, which was a Paprosky III case. Spacer dislocation might be attributed to the severe acetabular bone defect, and conservative treatments were performed. Two cases (7.7%) presented femoral spacer fractures. One of them was developmental dysplasia of the hip, with a small femoral canal that only one Steinmann pin was chosen for inner support. The other case might be caused by severe soft tissue contracture. Both of these two cases received stabilization with additional cerclage wires. The mean intervention between stages was 5.3 ± 3.7 (range 1.5-16) months. Finally, spacers were removed uneventfully for 24/26 hips, and revision THA was performed subsequently. One patient had resection arthroplasty because of severe bone defect of the femur. One patient was satisfied with the function of articulating spacer and unwilling to undergo revision THA. One patient developed acute PJI 5 years after the second-stage revision and underwent a debridement with the retention of the prosthesis. Twenty-five of 26 patients were treated successfully, reaching an overall success rate of 96.2%.

The mean follow-up time was 4.1 ± 2.2 (range 1-8) years. Hip Harris score before the first-stage revision was 40.9 ± 14.0 (range 16-71), and elevated to 81.2 ± 11.2 (range 55-99) (*p* < 0.05).

## Discussion

Two-stage revision technique is the “gold standard” in treating hip PJI. The eradication of infection through two-stage surgery reportedly could be greater than 90% in large numbers of researches [18–20]. Functional articulating spacers possess some advantages in maintaining soft tissue tension, avoiding muscle contracture, and enabling patients to restore through bearing partial weight after surgery. However, articulating spacers are not always perfect. Many studies have reported high incidence rates of spacer dislocation and fracture [21–23]. What is more, preoperative acetabular bone loss would emerge, and subsequent removal of acetabular components would increase spacer instability, leading to spacer dislocation or acetabular wear. Our study aimed to report early outcomes of augmented antibiotic-loaded cement spacer and to assess the subsequent occurrence of complications and related risk factors.

Spacer dislocation is not a rare complication of articulating spacer, and could lead to hip pain, limited range of motion (ROM), lower limbs shortening, and soft tissues contracture. Some severe cases could also cause sciatic nerve paralysis. The most common reason of spacer dislocation is smaller anteversion of spacer implantation and severe acetabular bone defect. Other reasons include mismatching between spacer head and acetabular size (bigger or smaller head), abnormal spacer head-neck ratio and offset, severely damaged soft tissues (gluteus medius muscle), instable fixation of proximal spacer, and poor compliance postoperatively [24–26]. Reported incidence rate of spacer dislocation is up to 10-20% [25–27]. All patients in our current study had different degrees of acetabular bone defect and were treated with augmented antibiotic-loaded cement spacer. The rate of spacer dislocation was only 3.8% (1/26), which was a Paprosky III case. The rate of spacer dislocation in our study was much lower than the previously reported data. However, due to the small sample size, the results require further verification, based on a large sample size.

Another common complication is spacer fracture and its reported incidence rate was 3.4-10% [23, 28, 29]. Common causes of spacer fracture include unsubstantial internal support of spacer, overweight bearing after surgery, and excessive antibiotic concentration (influence cement strength). Two cases (2/26, 7.7%) in our study developed spacer fracture, a similar figure to previously reported value. One of the two cases was developmental dysplasia of the hip. Femoral canal was so small that only one Steinmann pin was able to be adopted for inner support. The other case had severe soft tissue contracture. Increased head-neck ratio and offset were achieved to restore lower limbs’ length as much as possible during the second-stage revision operation. Therefore, spacer fractured due to overweight bearing without crutches.

Patients with acetabular bone defect of the medial wall or severe acetabular protrusion received temporary reinforcement from handmade acetabular spacers in our study. Hip joint activity was maintained without significantly increasing acetabular bone defect. Differences in Paprosky classifications (I, II, and III) between the first stage and second stage did not differ significantly (*p* > 0.05). As a result, the handmade acetabular spacer could prevent the femoral spacer from falling into the medial wall or prevent increases in acetabular protrusion, which avoided dislocation for spacer protrusion in the second stage. Besides, handmade acetabular spacer could restore hip offset and reduce spacer dislocation caused by offset deficiency. Hip Harris score was increased more frequently in the second stage than in the first stage. All of the results demonstrated that the application of antibiotic-loaded cement spacer might be an effective way to improve PJI for patients with acetabular bone defects. In the study of Ebied et al., hips’ reconstruction through the combination of tantalum metal augments (TMAs) and antibiotic-loaded impaction grafting was performed for 24 patients with combined segmental and cavitary acetabular defects. The rate of eradicating infection in that study was 97%, which was similar to our figure [30]. Compared to metal ones, cemented materials may reduce prosthesis-related complications [31]. In consideration of differences between cemented and uncemented materials, orthopedic doctors should carefully select antibiotic-loaded materials based on clinical experience and patients’ conditions and requirements.

Limitations in this study should be noticed. Firstly, the sample size was not large enough to obtain high statistical power, and present treatment outcomes were not compared to those of other antibiotic-loaded treatments. Secondly, the potential mechanism of periprosthetic joint infection was not investigated. Thirdly, interactions between augmented antibiotic-loaded cement spacer and other factors influencing periprosthetic joint infection were not detected either. In addition, controversy still exists over the application of cement prosthesis in hip arthroplasty. Cement prosthesis can make prosthesis fixed more firmly and stably, with a low incidence of periprosthetic fracture, infections, and complications. Moreover, some studies suggested that cement prosthesis might cause arrhythmia, oxygen saturation reduction, blood pressure drop, and other cardiovascular complications during the operation, and that it was difficult to repair whenever needed [31–33]. However, due to the limited study period, the occurrence of cardiovascular complications in our study population was not investigated. Therefore, further well-designed studies with large sample sizes should be performed to verify and improve our findings.

## Conclusion

In conclusion, the use of augmented antibiotic-loaded cement spacer could achieve fine clinical outcomes in PJI patients with acetabular bone defect. Hip joint activity was maintained with improved stability and decreased iatrogenic bone defect from acetabular wear.

## Data Availability

The datasets used and/or analyzed during the current study are available from the corresponding author on reasonable request.

## Abbreviations

PJI: Periprosthetic joint infection
THA: Total hip arthroplasty
ROM: Range of motion
